# Estimation of the Minimal Rift Valley Fever Virus Protective Neutralizing Antibody Titer in Human Volunteers Immunized with MP-12 Vaccine Based on Protection in a Mouse Model of Disease

**DOI:** 10.4269/ajtmh.22-0356

**Published:** 2022-09-19

**Authors:** Douglas M. Watts, Jonna L.B. Westover, Pedro M. Palermo, Kevin W. Bailey, John C. Morrill, George E. Bettinger, Thomas P. Monath, Darci R. Smith, Clarence J. Peters, Phillip R. Pittman, Jeanette Orbegozo, Brian B. Gowen

**Affiliations:** ^1^ Department of Biological Sciences and Border Biomedical Research Center, The University of Texas at El Paso, University of Texas at El Paso, El Paso, Texas;; ^2^ Institute for Antiviral Research and Department of Animal, Dairy, and Veterinary Sciences, Utah State University, Logan, Utah;; ^3^ Department of Microbiology and Immunology, University of Texas Medical Branch at Galveston, Texas;; ^4^ Crozet BioPharma LLC, Devens, Massachusetts;; ^5^ Department of Microbiology and Immunology, Naval Medical Research Center, Biological Defense Research Directorate, Fort Detrick, Maryland;; ^6^ Department of Pathology and Department of Microbiology and Immunology, University of Texas Medical Branch at Galveston, Texas;; ^7^ Department of Clinical Research 2 U.S. Army Medical Research Institute of Infectious Diseases (USAMRIID), Frederick, Maryland

## Abstract

The Rift Valley fever virus (RVFV) MP-12 vaccine is a promising human and veterinary vaccine. Although the vaccine elicited neutralizing antibody (nAb) in human volunteers, the minimal antibody titer that is needed to afford protection is unknown. Therefore, this study was conducted to determine the minimal nAb titer elicited by the RVFV MP-12 vaccine in human volunteers that protected mice against lethal RVFV challenge as a surrogate assessment of the protective efficacy of the vaccine. Among volunteers who were vaccinated with the MP-12 vaccine during a phase II trial, sera with antibody titers of 1:20 collected 5 years post-vaccination (PV), 1:40 titer collected 2 years PV, and 1:80 titer collected 1 year PV was passively transferred to groups of BALB/c mice. Blood samples were obtained 1 day after passive transfer to determine the RVFV neutralizing nAb titer before challenge with pathogenic RVFV (strain ZH501). Our results indicated that 1 day after passive transfer of the immune sera, an approximate 4-fold reduction in circulating nAb titers was detected in the mice. The presence of RVFV nAb titers in the range of 1:5 to 1:20 were generally protective (75–100% survival). These results suggested that circulating titers of 1:5 or higher offer a high degree of protection by MP-12-elicited antibody in human volunteers. Also, the findings highlighted the value of using the BALB/c mouse RVFV challenge model as a surrogate for evaluating the protective nAb responses elicited by MP-12 and possible use for evaluating the efficacy of other RVFV vaccine candidates.

## INTRODUCTION

Rift Valley fever virus (RVFV) causes devastating epizootics of Rift Valley fever (RVF) disease among domestic ruminants and epidemics among humans exposed to infected mosquitoes or come in contact with blood from infected ruminants.^1^^–^^3^ Several studies have indicated that RVF can be prevented in domestic animals by the use of vaccines to elicit neutralizing antibody (nAb) and that vaccination is the best approach for controlling RVF outbreaks.^4^^–^^8^ Although a human RVFV vaccine has not been approved, a formalin inactivated candidate vaccine designated as NDBR 103 was developed and evaluated clinically.^4^^,^^9^^–^^11^ This candidate vaccine was used to vaccinate 500 human volunteers and 963 United Nations soldiers using a three-dose regimen that resulted in seroconversions in most volunteers.^12^^,^^13^ The NDBR 103 vaccine candidate was modified by the Salk Government Services Division and designated TSI GSD 200 and was evaluated as a formalin-inactivated vaccine under an approved Investigational New Drug (IND) by the U.S. Army Medical Research Institute of Infectious Diseases (USAMRIID).^9^ The TSI-GSD-200 vaccine elicited 80% plaque reduction neutralizing (PRNT_80_) antibody titers of 1:40 or greater among 90% (540 of 598) of the volunteers given three 1.0-mL doses subcutaneously on days 0, 7, and 28 post-vaccination (PV).^9^ Although the TSI-GSD-200 vaccine was safe and immunogenic, it required a boost at 6 months, and subsequent periodic boosts were believed to be required to sustain protective antibody titers.^9^ Although the antibody titer required to afford protection among the vaccinees was unknown, the passive transfer of antibody elicited by the TSI-GSD-200 vaccine resulted in a titer as low as 1:12 that protected hamsters against a lethal RVFV challenge.^14^ Therefore, a vaccine-elicited RVFV antibody titer of 1:40 or more was judged to be protective for personnel who would be at risk to infection with pathogenic RVFV, including laboratory workers.^15^ Although inactivated vaccines elicited protective nAb, the shorter-term immunity and requirement for booster vaccinations makes their use difficult in resource-limited RVFV enzootic countries in Africa.^16^ Therefore, efforts to develop an improved vaccine for humans began to focus on live-attenuated vaccines that generally elicit longer-lasting immune responses without the requirement for booster vaccinations.^17^

Among several RVFV vaccine candidates under development, the RVFV MP-12 live-attenuated virus vaccine is among the most promising, both as a human and a veterinary vaccine.^8^ The vaccine was derived by 12 serial mutagenesis passages (MP) of ZH548 RVFV human isolate in human diploid MRC-5 cells in the presence of the chemical mutagen, 5-fluorouracil (5FU).^18^ The MP-12 vaccine is thermostable for 18 years or more after storage at −35°C, suggesting that it is suitable for stockpiling purposes.^19^^–^^21^ The attenuation of MP-12 was shown to be caused by mutations in the S, M, and L RNA segments, but that the mutations in the M and L segments confer stronger attenuation than those in the S segment. These mutations indicated that the risk of MP-12 reverting to virulence was low, which was supported by the finding that multiple attenuating mutations were effective in combination. Extensive evaluations demonstrated that MP-12 was safe and efficacious in laboratory animals and domestic ruminants, including lack of neurovirulence in nonhuman primates and safety for pregnant ruminants.^8^ Also, experimental studies indicated that the sporadic viremia produced in animals and humans by the vaccine was less than 3.0 log_10_ plaque-forming units/mL and therefore avoided the risk of transmission to mosquitoes.^19^^,^^20^^,^^22^^–^^24^ These data were used to obtain an IND for conducting phase I and II clinical trials that indicated that the vaccine was safe, well-tolerated, and immunogenic in healthy volunteers.^19^^,^^20^ Among 43 recipients of the MP-12 vaccine in the phase I trial who received a single dose, IgM and IgG antibodies were demonstrated in 40 (93%) and 28 (82%) of 34 of these vaccine recipients who were available 1 year later remained seropositive (PRNT_80_ = ≥ 1:20).^19^ In the phase II trial, only a single dose via the subcutaneous or the intramuscular route was required to elicit an immune response with sustained nAb ≥ 1:20 in 87% to 93% of the volunteers for 12 months and in eight of nine of the volunteers who were available at 5 years PV.^21^ Although transient adverse effects were reported in the volunteers, including headache, fatigue, nausea, fever, and local tenderness, they were well tolerated, causing mostly mild reactions without sequelae. The results suggested that the vaccine caused, at most, only a low-level viremia. Attempts failed to detect virus in blood samples obtained from the vaccinees by direct plaque assay; however, during the first 14 days after vaccination, nine MP-12 virus isolates were recovered from plasma of five of 19 vaccinees by sequential passage in Vero cells.^20^ Evidence based on RNA sequencing of the MP-12 isolates indicated that there were no reversions of any of the MP-12 mutations to those of the parent virulent virus (strain ZH548).^20^ These results suggested that the MP-12 vaccine candidate was genetically stable during in vivo replication. Although the safety and immunogenicity observations indicated that the MP-12 was a promising human vaccine, the minimal protective antibody titer elicited by this vaccine is unknown.

Several studies involving laboratory animals, domestic ruminants, and humans identified nAb as the likely correlate of protection against RVF.^2^^,^^7^^,^^8^^,^^14^^,^^25^^–^^31^ Although antibody affords protection against RVF, estimates of antibody titers at the time of actual challenge studies with virulent RVFV were not done. Also, the studies were not designed appropriately to determine the minimal protective antibody titer response. Estimates of the protective antibody titer elicited by human RVFV vaccine candidates cannot be done by experimental challenge of vaccinated humans with virulent RVFV, and field trials designed to estimate protective efficacy during epidemic of RVF in enzootic settings have not been conducted. The lack of an understanding of the minimal antibody titers that afford protection represents a major gap in the development and evaluation of both human and veterinary RVFV vaccine candidates. If an immune correlate reasonably indicating protection and clinical benefit could be defined, it may be possible to register a vaccine using this accelerated pathway, with commitment post-marketing for a field study should the opportunity arise (https://www.fda.gov/drugs/information-health-care-professionals-drugs/accelerated-approval-program). Therefore, the objective of this study was to estimate the minimal protective nAb titer elicited by the MP-12 vaccine by passively transferring serum samples from vaccinated human volunteers into mice and assessing nAb titers just before lethal RVFV challenge of the mice. The lowest nAb titer that protected the animals was interpreted as an estimate of the minimal protective efficacy of the vaccine.

## MATERIALS AND METHODS

### Virus.

The molecular clone of RVFV, strain ZH501, was obtained from Dr. Stuart Nichol (CDC, Atlanta, GA). The infectivity titer of the RVFV stock was 1.1 × 10^8^ plaque-forming units (PFU)/mL). The stock virus was derived from a single passage in BSR-T7/5 cells, three passages in Vero E6 cells and clarified by centrifugation. The BSR-T7/5 cells are baby hamster kidney-derived cells that express T7 RNA polymerase. They were kindly provided by Dr. K. Conzelman (Max-von Pettenkofer-Institut, Munchen, Germany). The ZH501 virus was diluted in sterile medium, and approximately 100 PFU was inoculated by the subcutaneous (SC) route on the right side of the abdomen of mice in a volume of 0.1 mL.

### Mice.

Male and female 6- to 8-week-old BALB/c mice (Charles River Laboratories, Wilmington, MA) were acclimated for 6 days before receiving the human MP-12 antibody-positive sera samples and fed Harlan Laboratory Block and tap water ad libitum. All animal procedures used in this study were in compliance with guidelines set by the U.S. Department of Agriculture and the Utah State University Institutional Animal Care and Use Committee.

### Serum samples.

Human sera were provided by the USAMRIID. The samples were provided as frozen aliquots of 0.5 to 2.0 mL and were obtained from civilian and military volunteers who had been vaccinated with a placebo or a single shot of 1 mL of the MP-12 vaccine via the intramuscular (IM) route during the phase II vaccine trial.^20^ The serum samples were anonymized and declared to be exempt from informed consent if used exclusively for research purposes by the USAMRIID and the University of Texas at El Paso’s Institutional Review Boards. The serum samples selected for this study included one obtained from volunteers at 1 year PV with a PRNT_80_ nAb titer of 1:80, one sample obtained at 2 years PV with a titer of 1:40, and a sample obtained at 5 years PV with a titer of 1:20 as being representative of the lowest titers elicited by the MP-12 vaccine in the volunteers. Also, 4 nAb-negative samples obtained from a volunteer before vaccination were pooled and used as a negative control. Although the nAb titers had been determined previously during the vaccine trial,^20^ the titers were confirmed before use in this study by testing 2-fold dilutions of the samples using the PRNT_80_ procedures described subsequently.

### Experimental design.

The testing of human serum samples to determine the minimal protective antibody titer elicited by the MP-12 vaccine candidate involved the passive transfer of the samples to mice followed by challenge of the mice with a lethal dose of virulent RVFV. Before the passive transfer with the human serum samples, mice were assigned to each experimental group (*n* = 4 per group) so that the average weight per group across the entire experiment varied by < 1 g and equal numbers of male and female animals were included per group. Each of three human serum samples with known nAb titers of 1:20, 1:40, and 1:80 were passively transferred to BALB/c mice by IP injection with 0.2 mL per mouse. In addition, one group of four mice received negative control serum. Whole blood samples containing approximately 150 µL were collected from each mouse by submandibular vein puncture 24 hours post-transfer. The blood samples were centrifuged for 15 minutes at 3000 × *g*, and the serum fraction was removed and stored at –20°C until tested by PRNT. The animals were allowed to recover for 2 hours before transfer to the biosafety level 3+ (BSL3+) facility. After the 2-hour recovery period, the mice were challenged via the SC route with 100 PFU of RVFV ZH501, the dose at which approximately 90% of the challenged mice succumb (LD_90_ based on titration of the virus stock in BALB/c mice). Two control mice were SC injected with minimal essential medium as sham-infected normal controls. The animals were observed for 21 days for morbidity and mortality. The prechallenge mouse serum samples were tested by PRNT to determine the RVFV nAb titers. The lowest nAb titer that protected the mice was the minimal protective level of antibody.

### Plaque reduction neutralization test.

Sera were heat inactivated at 56°C for 30 minutes and tested for nAb to the MP-12 virus as described previously.^20^ Briefly, equal volumes of 2-fold dilutions ranging from 1:5 or 1:10 through 1:1280 of each sample were prepared in minimal essential medium and incubated overnight at 4°C with an equal volume of 50 to 100 PFUs of the MP-12 virus. On the following day, 50 µL of the virus–sera mixture were inoculated in duplicate onto a confluent monolayer of Vero E6 cells grown in 24-well plates. Cultures and inocula were incubated for 1 h at 37°C and 5% CO_2_. A mixture of 1% SeaKem agarose (VWR) with an equal volume of 2× Eagle’s basal medium with Earle’s salt, HEPES, sodium bicarbonate, 8% fetal bovine serum, and 1% penicillin/streptomycin and L-glutamine (Thermo Fisher Scientific) was prepared and 0.5 mL was overlaid onto each cell culture. The agarose overlay was allowed to solidify, and then the cultures were incubated for 3 days at 37°C and 5% CO_2_. At 3 days post-infection, cultures were stained with a 0.33% neutral red solution and incubated for 4 to 6 hrs to identify PFUs. The PFUs were counted and the dilution of serum that reduced the number of plaques relative to the negative human serum control by 80% was considered to be the nAb titer.

### Assessment of viral loads in organs of selected mice that succumbed to lethal RVFV infection.

Tissue samples were collected from selected mice that succumbed to RVFV challenge, and virus titers were assayed using an infectious cell culture assay as previously described.^32^ Briefly, a specific volume of tissue homogenate was serially diluted and added to triplicate wells of Vero 76 cell monolayers in 96-well microtiter plates. The viral cytopathic effect was determined 5 days after plating and the 50% endpoints were calculated as described.^33^ The lower limit of detection was 2.1 log_10_ 50% cell culture infectious dose per gram of tissue. In samples presenting with virus below the limits of detection, a value representative of the limit of detection was assigned for statistical analysis.

### Statistical analysis.

The Mantel–Cox log-rank test was used for analysis of Kaplan–Meier survival curves using Prism 8.3 (GraphPad Software).

## RESULTS

Among human volunteers vaccinated with the MP-12 vaccine, selected antibody positive sera samples were used to determine the minimal nAb titer that afforded protection to mice (Table 1). The serum samples included one with a PRNT_80_ titer of 1:20 that was taken 5 years PV, one with a 1:40 titer that was taken 2 years PV, and a sample with a 1:80 titer that was taken one year PV. On transfer of the sera to each group of 4 mice, the human serum sample with a 1:20 titer resulted in a prechallenge titer of 1:5 in the serum of all 4 mice. The 1:40 titer human serum sample decreased to 1:10 in the serum of 3 mice and 1:20 in the fourth mouse. The 1:80 titer human serum sample decreased to 1:20 in the serum of all 4 mice at 24 hours post-transfer. After challenge of each group of four mice with virulent RVFV ZH501, three of four mice (75%) that had a prechallenge nAb titer of 1:5, three mice that had prechallenge nAb titer of 1:10, and one with a 1:20 titer (100%) and three of four mice (75%) with a prechallenge nAb titer of 1:20 were protected. Thus, the minimal protective antibody titer that protected 75% of mice against an LD_90_ challenge dose of RVFV was 1:5 or greater.

**Table 1:** Summary of the MP-12 human volunteer sera samples nAb titers before passive transfer into mice, nAb titers 24 hours after passive transfer, and protection from challenge with pathogenic RVFV ZH501

Mouse group no.	Animal identification nos.	Sex	Human volunteer serum codes	Human sera RVFV nAb titer^1^	nAb titers in mouse sera^4^	Days postchallenge animals expired
2	289	M	–	–	**≤ 5**	Sham-inoculated^2^
2	290	F	–	–	**≤ 5**	Sham-inoculated^2^
1	291	M	G0004^3^	**≤** 10	**≤ 5**	4
1	292	M	G0005^3^	**≤** 10	**≤ 5**	6
1	293	F	G0006^3^	**≤** 10	**≤ 5**	4
1	294	F	G0007^3^	**≤** 10	**≤ 5**	5
3	295	M	G2185	20	5	Survived
3	296	M	G2185	20	5	Survived
3	297	F	G2185	20	5	Survived
3	298	F	G2185	20	5	19
5	299	M	G2156	40	10	Survived
5	300	M	G2156	40	10	Survived
5	301	F	G2156	40	20	Survived
5	302	F	G2156	40	10	Survived
7	303	M	G2136	80	20	9
7	304	M	G2136	80	20	Survived
7	305	F	G2136	80	20	Survived
7	306	F	G2136	80	20	Survived

F = female; M = male; nAb = neutralizing antibody; RVFV = Rift Valley fever virus.

^1^ nAb titer of human sera samples before passive transfer to mice.

^2^ Control mice did not receive serum and were sham inoculated with minimum essential media.

^3 ^Pooled antibody-negative sera samples obtained from human volunteers before receiving the RVFV MP-12 vaccine and the pooled sera was transferred to each of four mice that were challenged with a virulent strain of RVFV to demonstrate that the challenge dose caused a lethal infection of the mice.

^4^ Reciprocal nAb titer (PRNT_80_) 24 hours after passive transfer of sera samples to mice.

**Figure 1.**
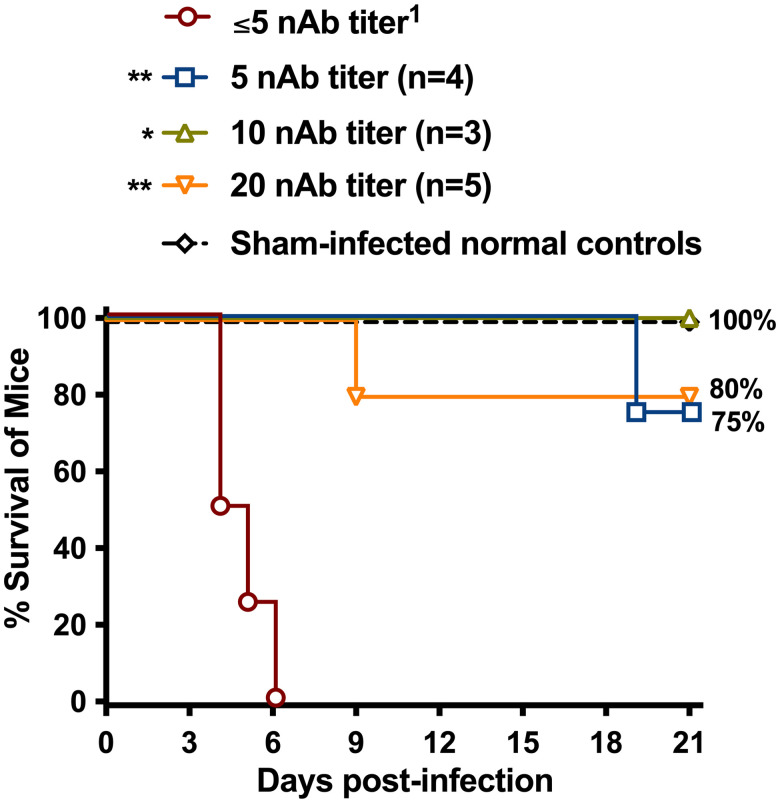
Survival outcome of BALB/c mice treated with human immune or nonimmune sera 26 hours before subcutaneous challenge with virulent Rift Valley fever virus (RVFV) ZH501. Animals in each group (*n* = 4) were administered human sera samples with defined MP-12 neutralizing antibody (nAb) titers. The survival groups are shown based on the nAb titers measured in the mice 24 h after passive transfer. ** *P* < 0.01 compared with animals that received the control human nAb-negative serum. ^1^Reciprocal human serum neutralizing antibody titer (PRNT_80_) measured in the mice just before RVFV infection.

The survival curves for each group of mice treated with the different nAb titers and challenged with virulent RVFV are presented in Figure 1. All four mice that received the nAb-negative control sera succumbed to infection by day 6 postchallenge (PC). In contrast, mice that received the nAb-positive sera were significantly protected (*P* < 0.01) against lethal disease. As shown in Table 1, a single animal with a nAb titer of 1:5 at the time of RVFV challenge succumbed on day 19 PC. This animal (#298) was suffering from delayed-onset neurologic disease. Unexpectedly, a single animal (#303) with a 1:20 nAb titer at the time of challenge also succumbed from neurologic manifestations on day 9 PC.

**Figure 2.**
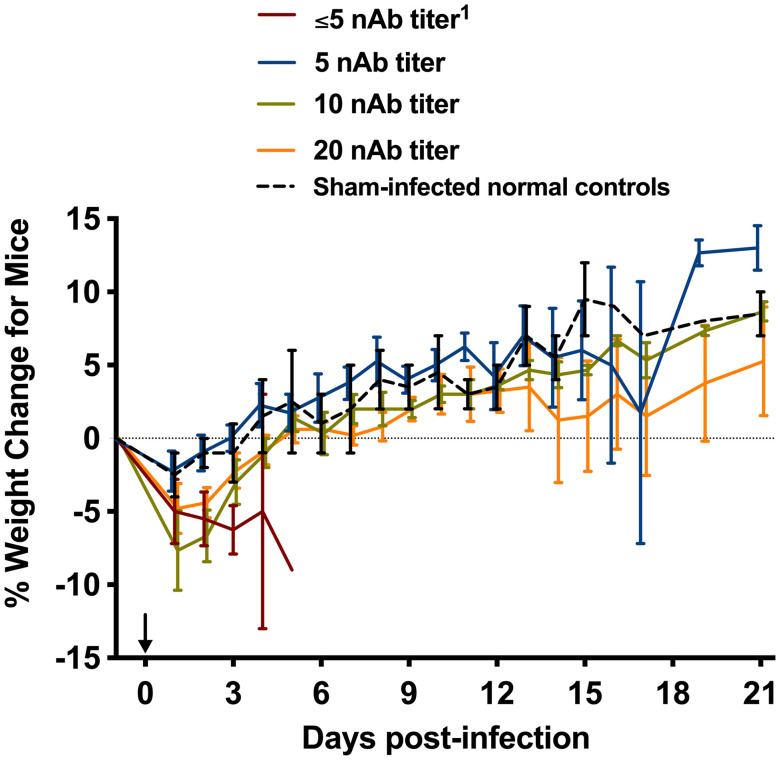
The percent daily average weight change for each group of four BALB/c mice that received human immune or nonimmune sera 26 hours before subcutaneous challenge with virulent Rift Valley fever virus (RVFV) ZH501. The data shown are grouped by the resulting nAb titers measured in the mice 24 h postpassive transfer and are represented as the neutralizing antibody (nAb) titer group mean and standard error of the percent change in weight of surviving animals relative to their starting weights on the day of treatment (d –1) and then measured daily. The arrow indicates RVFV challenge on day 0.^1^^ ^Reciprocal human serum nAb titer (PRNT_80_).

The lack of protection observed in this single mouse administered human sera with 1:80 nAb titer was surprising, and thus we retrospectively collected tissues from the frozen carcasses of mouse #303 and other selected animals for viral titer analysis. As shown in Figure 2, animal #303 had high viral loads in the liver and brain tissue. By comparison, animal #291, which was administered nonimmune human serum, had similarly high viral loads in all three collected tissues when it succumbed on day 4 PC. Animals 289 (sham-infected control) and 304 (administered 1:80 nAb titer human serum and survived) had undetectable virus titers in all samples tested.

**Figure 3.**
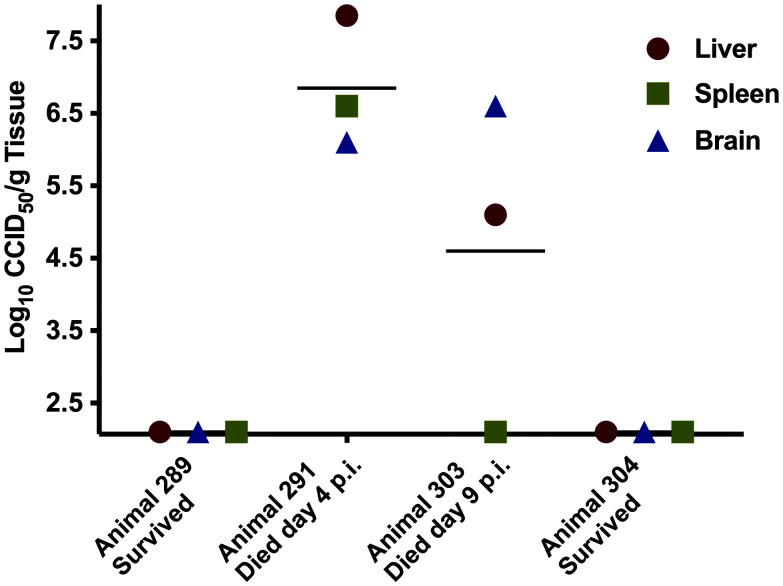
Rift Valley fever virus (RVFV) titers in tissues of selected mice at their respective time of death or euthanasia. The four animals were selected retrospectively after the completion of the study for analysis of virus titers in liver, spleen, and brain. Animal #289 was a sham-infected control, animal #291 received nonimmune serum and succumbed to acute RVFV disease on day 4 postchallenge, animal #303 received nAb-positive serum and unexpectedly succumbed to later-onset neurological disease, and animal #304 received nAb-positive serum and survived RVFV challenge with no evidence of disease. The x-axis defines the assay limits of detection.

The percent daily weight change of the mice relative to their starting weights when the animals received immune or nonimmune sera were consistent with the survival data (Figure 3). The mice that received the nAb-negative sera and ultimately succumbed after challenge with RVFV abruptly lost weight as they approached the terminal stages of disease. The animals that survived the RVFV challenge generally gained weight at a trajectory similar to that of the sham-infected normal control mice.

## DISCUSSION

Among the existing human and domestic ruminant RVFV vaccine candidates, the live-attenuated MP-12 vaccine is the only human vaccine in advanced development that has been evaluated extensively and found to be safe and efficacious in laboratory animals and domestic ruminants and to be well tolerated and immunogenic in human volunteers.^8^^,^^19^^–^^21^ Our findings in this study extended observations for the immune response of humans to the vaccine by demonstrating that the passive transfer of serum from MP-12-immunized individuals resulting in a passive nAb titer in mice as low as 1:5 afforded protection to 75% (3 of 4) of mice and a titer of 1:10 to 1:20 protected 89% (8/9) of the mice following challenge with a lethal dose of virulent RVFV. Consistent with our findings, Niklasson et al. showed that human sera samples with an antibody titer of 1:12 from volunteers vaccinated with an inactivated RVFV vaccine and transferred to hamsters protected against lethal RVFV challenge.^14^ Also, immunization of non-human primates with MP-12 revealed that a titer of 1:40 protected against challenge with virulent RVFV, but these studies were not designed to determine the minimal antibody titers that afforded protection.^34^ Overall, our findings are supportive of previous observations that showed nAb to confer protection against RVFV infection of human and domestic ruminants and demonstrated the high protective efficacy of low concentrations of antibody following a single dose of the MP-12 vaccine candidate.^2^^,^^7^^,^^8^^,^^14^^,^^25^^–^^31^

Our study using passive immunization in a mouse model is the first to estimate the minimal protective antibody level elicited by MP-12 in human volunteers. If our findings of a minimal 1:5 to 1:20 protective antibody titer are extrapolated to MP-12 clinical trials, then all but one of 62 volunteers involved in the phase I and II trials developed protective antibody titers on or before day 28 PV.^19^^,^^20^ The findings during the phase II vaccine trial were more extensive in that 18 of 19 (95%) volunteers administered a single 1-mL injection of MP-12 (1 × 10^5^ pfu), including 9 women and 9 men achieved a PRNT_80_ that ranged from 1:60 to 1:1920 by PV day 28, far exceeding the estimated minimal protective titer. The other vaccinee developed a 1:10 titer, which may be minimally protective. At one year PV, the antibody titer for that one subject was ≤ 1:10, but the remaining 18 subjects’ titers were retained at 1:60 to 1:1280. At 2 years PV, the 13 available volunteers had antibody titers that ranged from 1:30 to 1:240. The titers for 9 volunteers available at 3 years PV ranged from 1:20 to 1:1280, and at 4 years PV, the titers for 8 available volunteers ranged from 1:30 to 1:480, and at year 5 PV for 9 available volunteers, the antibody titers ranged from 1:15 to 1:480. These data strongly suggest that after 5 years many of the MP-12 elicited antibody titers among the human volunteers in the phase II trial were higher than the minimal antibody titer of 1:5 to 1:20 required to afford protection against a lethal challenge dose of virulent RVFV in Balb/c mice. Furthermore, the humoral immune response elicited by the MP-12 vaccine that remained detectable for several years in humans, was also found to afford protection to vaccinated non-human primates for 6 years.^35^ These are the only results showing that a single dose of the MP-12 vaccine elicited protective antibody in human volunteers lasting for 5 or more years.

Severe RVF disease in humans and livestock is characterized by acute-onset hepatitis and delayed-onset encephalitis. These manifestations are reproduced in the BALB/c RVF mouse model which is highly susceptible to RVFV infection.^36^^–^^39^ The 2 mice that received antibody positive sera and succumbed on days 9 and 19 PC with virulent RVFV showed evidence of neurologic disease suggesting that these animals succumbed from a late-onset encephalitis. The mouse that was moribund on day 9 had the most virus in the brain, with reduced levels in the liver and no detectable virus in the spleen. However, with such a highly susceptible animal model it is important to note that even low levels of nAb were protective in the majority of mice. These results were consistent with observations from a previous study where some passively immunized hamsters died relatively late or up to 11 days after challenge with a lethal dose of RVFV.^14^ Similar observations among RVFV challenged immunized inbred rats suggested that the animals succumbed to encephalitis because of partially protective antibody.^40^ This phenomenon may be analogous to the treatment of another hemorrhagic fever (Argentine hemorrhagic fever caused by Junin virus), in which patients treated with convalescent plasma containing nAb were rescued from the viscerotropic infection, only to develop a late-onset neurologic syndrome (LNS).^41^ Notably, however, most of the patients that suffer from LNS do survive the disease. The observation of this phenomenon in the passively immunized mice in our study and in Junin patients, most likely would have been prevented by active vaccination that stimulated T cell responses needed to clear the viral infection.^42^

As a neglected zoonotic viral disease, RVF has long been recognized as a disease with a devastating impact on the health of humans and domestic ruminants in Africa, Madagascar, some Indian Ocean islands, and more recently in the Arabian Peninsula.^1^^–^^3^ The human health threat is further implicated by the possibility of global spread and the potential use of the RVFV as a bioterrorism weapon.^43^^,^^44^ Even though numerous studies have shown that both inactivated and live-attenuated vaccines can prevent RVF in domestic ruminants, and most likely among humans, efforts to develop and approve safe and efficacious RVF vaccines still remains a global need.^5^^,^^7^^,^^8^^,^^45^ The reasons vaccines are not available are due in part to the lack of advancing the development of promising vaccine candidates because of the lack of funding to obtain an understanding of the risk and the benefits needed to make a regulatory decision.^17^ Also, for some approved and promising veterinary vaccine candidates, an effective vaccination strategy is needed to use them safely and effectively.^17^^,^^46^

While the MP-12 live-attenuated vaccine is among the most advanced of the human vaccine candidates, it has not been approved for human use. The robust immunogenicity of this vaccine has been interpreted as an indicator of an efficacious vaccine, but protective efficacy studies have not been conducted via controlled field vaccine trials. Such trials may be difficult logistically since RVF epidemics do not occur on a regular pattern. Alternatives to the traditional pathway of a randomized controlled trial include the accelerated approval pathway and the Animal Rule. Accelerated approval would allow marketing of the vaccine with a post-marketing obligation to perform an efficacy study in the future, which might be done in the context of a large epidemic. This pathway requires acceptance of a biomarker or immune correlate reasonably predictive of clinical benefit. As an effort to identify such a correlate, we conducted a passive immunization study with sera from MP-12 vaccinated individuals in a lethal mouse challenge model. The results suggested that PRNT_80_ nAb titers as low as 1:5 elicited by a single dose of the MP-12 could afford protection in a highly susceptible mouse model of RVFV infection and disease. Also, the protection afforded was consistent with published studies indicating that nAb confers protection against RVF. Further studies to define an immune correlate by passive transfer of antibody and challenge of nonhuman primates with a well-characterized RVFV strain may offer a reliable approach for evaluating candidate human and veterinary vaccines.

As a possible limitation regarding the interpretation of the protective efficacy of the MP-12 candidate vaccine, estimates of the concentration of antibody elicited in human volunteers by the vaccine was based on PRNT_80_ analysis using the MP-12 vaccine virus in Vero E6 cells. Also, the concentration of antibody after passive transfer to mice was estimated before challenge with RVFV using the same procedure. As a result, it is possible that the concentration of nAb based on the use of the MP-12 virus in the PRNT_80_ assay may differ from the concentration measured using a pathogenic strain of RVFV. However, the results of previous experiments demonstrated that the neutralizing activity of antibody elicited by the MP-12 and the ZH501 strains of RVFV in sheep and nonhuman primates were comparable with less than 2-fold difference based on PRNT_80_ cross neutralization (J. C. Morrill, personal communications). The comparable neutralizing activity of the antibody most likely reflected the very low genetic diversity of approximately 5% based on the full genome sequences of 33 virus isolates that comprised the seven known RVFV lineages.^47^^–^^49^ As a result of the high degree of sequence conservation of the M segment genes that encode virion surface glycoproteins, antibodies elicited by a single strain of RVFV neutralized all circulating RVFV, implying that antibody elicited by a single vaccine would protect against all lineages of pathogenic RVFV.

While the results of this study further emphasized the critical role of vaccine-elicited nAb for protecting against RVF disease, the possible protective role of the innate and cellular immune response was not addressed. However, several studies have shown that the both the innate.^42^^,^^50^^–^^59^ and cellular immune response.^54^^–^^66^ Contributed to the protection of mice, rats, sheep, goats, and nonhuman primates against RVF disease.
